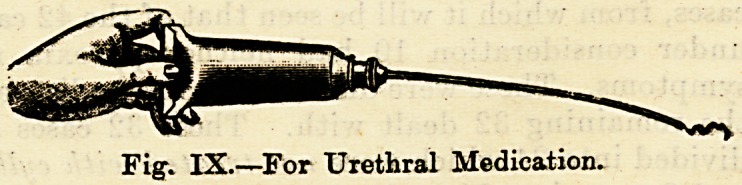# New Appliances and Things Medical

**Published:** 1906-10-20

**Authors:** 


### NEW APPLIANCES AND THINGS MEDICAL.

[We shall be glad to receive at our Office, 28 & 29 Southampton Street, Strand, London, W.C., from the manufacturers, specimens of all new preparations and appliances which may be brought out from time to time.]

### CONTINUOUS-FLOW SYRINGE (SUMNA).

(Messrs. R. Sumner and Company, Ltd., Lord Street, Liverpool.)

The importance of this instrument can scarcely be over-estimated. From the days when a perforated gourd was filled with salt and water to be used as a rectal injection—which is still in use by some African nations—it seems a long cry to a syringe of such perfection as the "Continuous-flow

Fig. I.—The "Sumna" Continuous-flow Syringe.

syringe." It indicates great advances, not only in the manufacture of these instruments, but in their immediate application to the prevention and cure of disease. To the midwifery nurse the enema syringe is an absolute essential. Crude methods of syringing or jerky, impulsive syringes are, however, fraught with so much danger that they are apt to do more harm than good, even when manipulated by persons who have some experience. The "Sumna" syringe, however, obviates any danger from intermittency or fitfulness. It ensures a careful and slow manipulation during which the force and pressure of the flow is regulated not by the hand of an excitable nurse, but by the automatic mechanism of elastic pressure. In this way an even current of hot or cold water may be applied without risk or danger, and the parts comfortably freed from septic matter. The liquid, to be effective, should flow slowly, so as to keep the neck of the womb constantly immersed in a pool of slowly flowing fluid, in this way allowing the necessary time for the temperature of the water to exert its effect, or for any remedy to be absorbed by the mucous membrane or surrounding parts. This syringe is made from the purest sheet india-rubber. The sinker is carefully covered, weighted, and protected by wire gauze from any gross material which is liable to clog or erode the valves. The whole instrument is capable of being taken to pieces, and completely sterilised with suitable disinfectants. Care should be taken, however, not to use corrosive or irritant disinfectants, or such as may spoil the rubber or corrode the vulcanite joints. For midwifery nurses the instrument with the continuous flow is fitted with vaginal and rectal pipes, and in price does not greatly exceed the commoner instruments, while its higher quality makes it cheaper in the long run. For the gynæcologist various fittings are

Fig. II.—Vaginal Tube with Groove.

made: the uterine tube, with groove for the back flow, or a thin uterine stem for gentle douching of the uterus. The instrument is also supplied with a junction for fitting the stopcock of Barnes's bags, and enabling them to be dis-

Fig. III.—Uterine Douche.

tended equably. To the syringe can also be attached Harrison's irrigator for the urethra—a most useful instru-

Fig. IV.—Harrison's Irrigator.

ment to the general practitioner. Nose and ear pieces for applying medicated douches, and one piece which, when connected, forms a gentle eye douche—most useful in

Figs. V. and VI.—Nose and Eye Fittings.

ophthalmic work are supplied. Considering the general practical utility of this instrument and its various connections, the "Sumna" syringe should be the constant companion of the general practitioner, especially where midwifery practice is extensive, and for the rough usage which that entails in a country district it is by far too valuable

Fig. VII.—Rectal Ointment-introducer.

Fig. VIII.—Uterine Ointment-carrier.

Fig. IX.—For Urethral Medication.

an instrument to be put up in so flimsy a box as is the case at present. In the present one the pieces are likely to be lost when the elastic gets worn, or case may get crushed and the syringe injured, thus causing great vexation and disappointment. This firm also supplies vulcanite parts for the application of ointments to some internal passages and organs. Rectal tubes are attached to collapsable ointment-carriers, forming a most useful and clean method of applying medicated lubricants. The apparatus for applying medicated ointments to the urethra will be of service in cases of gleet or urethritis. The uterine ointment-introducer will also be highly serviceable as a much-needed appliance in the practice of a country doctor.

## Figures and Tables

**Fig. I. f1:**
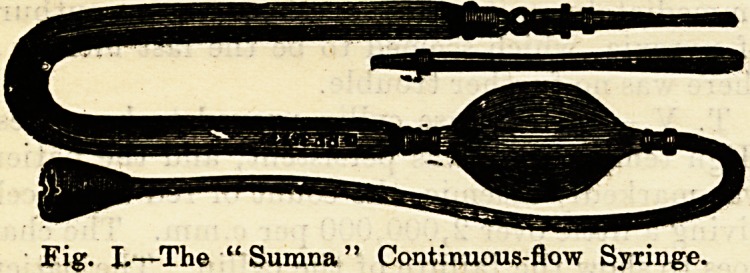


**Fig. II. f2:**
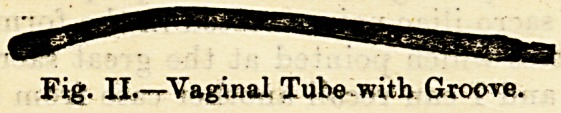


**Fig. III. f3:**
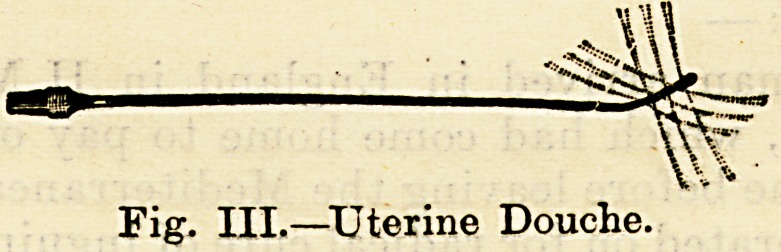


**Fig. IV. f4:**
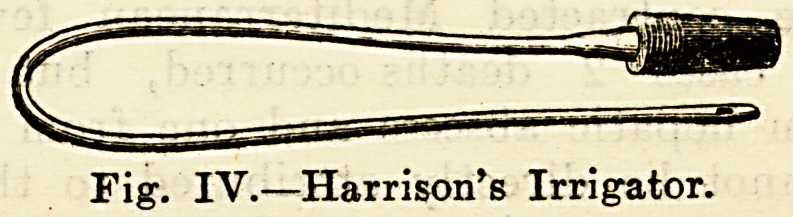


**Figs. V. and VI. f5:**
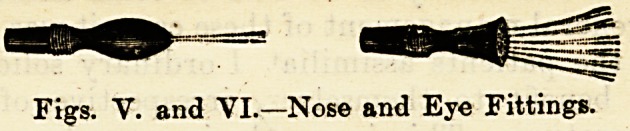


**Fig. VII. f6:**
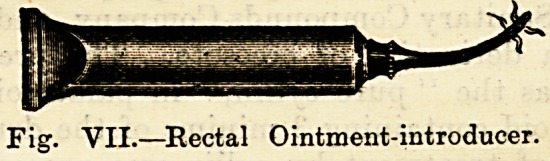


**Fig. VIII. f7:**
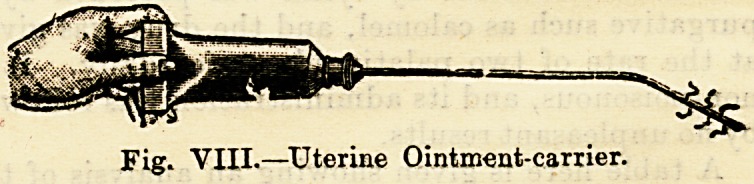


**Fig. IX. f8:**